# Expression of miR-210-3p as a Prognostic Marker for Development of Diabetic Neuropathy

**DOI:** 10.3390/metabo16010013

**Published:** 2025-12-23

**Authors:** Savelia G. Yordanova, Diana Nikolova, Zdravko Kamenov, Vera Karamfilova, Traykov Lachezar, Yavor Assyov, Tsvetan Gatev, Radka Kaneva, Olga Belcheva, Darina Kachakova, Veronika Petkova, Yavor Zhelev, Antoaneta Trifonova Gateva

**Affiliations:** 1 Faculty of Medicine, Department of Internal Medicine, 1431 Sofia, Bulgaria; 2 Faculty of Medicine, Department of Neurology, 1431 Sofia, Bulgaria; 3 Faculty of Medicine, Department of Medical Chemistry and Biochemistry, 1431 Sofia, Bulgaria

**Keywords:** miR-210-3p, diabetic neuropathy, corneal confocal microscopy, type 2 diabetes, microRNA, delta Ct

## Abstract

**Background/Objectives**: Diabetic neuropathy (DN) is one of the most common complications of type 2 diabetes mellitus (T2DM), involving complex metabolic, vascular, and epigenetic mechanisms. MicroRNA-210-3p (miR-210-3p), a hypoxia-responsive molecule, has been implicated in various diabetic complications, but its role in DN is not well defined. This study aimed to investigate the relationship between miR-210-3p expression, measured as delta Ct (ΔCt), and the presence and type of diabetic neuropathy, as well as correlations with corneal nerve parameters assessed by corneal confocal microscopy (CCM). **Methods:** Eighty patients with T2DM were stratified into four groups: no neuropathy, autonomic neuropathy, peripheral neuropathy, and combined neuropathy. Expression of miR-210-3p was quantified using RT-qPCR, and CCM was used to measure corneal nerve fiber density (CNFD), length (CNFL), and branch density (CNBD). **Results:** ΔCt values were significantly lower in patients with combined neuropathy compared to those without neuropathy, indicating higher miR-210-3p expression. Intermediate values were observed in autonomic and peripheral neuropathy groups. CCM parameters were significantly reduced in patients with DN. ΔCt was inversely correlated with neuropathy severity but positively associated with diabetes duration. **Conclusions:** These findings suggest that miR-210-3p may serve as a biomarker of nerve damage and cellular stress in diabetes, and that combining gene expression profiling with CCM could improve DN diagnosis and monitoring.

## 1. Introduction

Type 2 diabetes mellitus (T2DM) is a chronic, multisystemic disease that disrupts the glucose homeostasis and continues to emerge as a major health problem due to its pandemic rates of distribution. According to the International Diabetes Federation (IDF), nearly 1 in every 9 adults (589 million) is currently living with diabetes. Out of these, an estimated 252 million are not yet aware they have the condition, placing them at higher risk of serious complications and early death. These alarming findings suggest that many will be diagnosed when they already have one or more of the associated complications and will have missed opportunities to prevent or delay their onset [1].

Diabetic neuropathy (DN) refers to a heterogeneous group of medical conditions that affect the diabetic population with diverse clinical manifestations. It is the most common microvascular complication of T2DM as it affects almost 50% of patients after 10 years of disease duration, while it is estimated that 20% of diabetic patients are affected at the time of the diagnosis. Despite its high prevalence in the diabetic population, the diagnosis is often missed, as almost 50% of patients are asymptomatic [2]. As a clinical entity, DN represents a spectrum of neuropathic conditions with clinical manifestations ranging from peripheral neuropathy to various forms of autonomic dysfunction, including cardiovascular, gastrointestinal, genitourinary, sudomotor and pupillary dysfunction. By 2045, 783 million adults are projected to be living with diabetes worldwide with up to 350 million developing DN and its comorbidities. Individuals with diabetic peripheral neuropathy (DPN) may experience severe pain, loss of sensation, impaired balance, falls, ulcers, and amputations, all leading to reduced quality of life. Cardiovascular autonomic neuropathy (CAN) is a dreaded complication, as it can exacerbate cardiovascular disease and contribute to heart failure and sudden cardiac death. DPN alone accounts for over $10 billon of annual healthcare costs and more than one-fourth of the total direct medical cost of diabetes [3].

DN results from a complex interaction between hyperglycemia, dyslipidemia, and insulin resistance. Hyperglycemia activates multiple biological pathways, such as the polyol, glycolysis, hexosamine, and advanced glycation end product pathways, leading to oxidative stress, mitochondrial damage, and inflammation. These processes result in endoplasmic reticulum stress, deoxyribonucleic acid (DNA) damage, and an increased inflammatory response. Persistent mild inflammation, caused by ongoing stress or dysfunction, and increased levels of glucose, lipoproteins, and oxidative and glycated proteins altogether contribute to nerve damage [4].

Considering the presented data, including the progressively disabling course of diabetic neuropathy, the reduction in quality of life and the increasing financial burden, new, reliable biomarkers are necessary for the early diagnosis and prevention of this complication.

In light of recent studies, microRNAs (miRNAs) are now broadly investigated in the pathogenesis of different diseases. These small non-coding RNAs regulate gene expression through base-pairing with mRNA targets and participate in essential cellular processes including proliferation, metabolism, apoptosis, and intercellular communication. Their dysregulation has been linked to numerous conditions such as cancer, neurodegenerative, autoimmune, cardiovascular, and endocrine diseases. Since specific miRNA patterns can reflect early pathological changes, they hold promise as biomarkers for disease onset, progression, and treatment response [5].

It has been discovered that some miRNAs are regulated by hypoxia, and miR-210 is the leading and most consistently hypoxia-induced miRNA. The mechanism by which this occurs, as suggested by Crosby et al., stems largely from the induction of miR-210 by HIF1α (Hypoxia-Inducible Factor 1 alpha) binding to its hypoxic response element [6]. Hypoxia is a feature of several pathological conditions, and a number of them, including malignancies, atherosclerosis, myocardial infarction and diabetic heart failure, are states in which miR-210 contributes to cellular hypoxic response [7].

MiR-210-3p, the mature and functionally active strand of miR-210, regulates diverse biological pathways such as cell cycle control, survival, differentiation, angiogenesis, mitochondrial metabolism, DNA repair, and immune signaling [8]. Its dysregulation has been implicated in several diabetes-related conditions, including gestational diabetes mellitus (GDM), type 2 diabetes mellitus (T2DM), and diabetes-associated tissue dysfunctions, through mechanisms involving inflammation, insulin resistance, vascular impairment, and hypoxia. In GDM, increased miR-210-3p is found to impair pancreatic β-cell viability and function [9], while its role in obesity focuses on obese adipose tissue microenvironment [10,11].

Collectively, these findings position miR-210-3p as a possible critical player in the onset and progression of various diabetes-related pathologies. The established connections between this particular miRNA, hypoxia, vascular and endothelial injury provides valuable insight regarding diabetic neuropathy. Further investigation into miR-210-3p as a therapeutic target and diagnostic biomarker is warranted, especially in the context of personalized medicine for diabetes management. The aim of our study is to determine if certain changes in miR-210-3p expression could serve as a prognostic marker for diabetic neuropathy (both peripheral and autonomic), potentially aiding in early detection and risk stratification.

## 2. Materials and Methods

### 2.1. Study Design

This was an analytical, observational, cross-sectional, monocentric study conducted at the Endocrinology and metabolic disorders clinic in Alexandrovska hospital, Sofia, Bulgaria. A total of 80 patients with a confirmed diagnosis of type 2 diabetes mellitus (T2DM) were recruited. Inclusion criteria were age between 18 and 75 years, diagnosis of T2DM for at least 1 year, and ability to provide informed consent. Exclusion criteria included concomitant ocular diseases (e.g., keratoconus, glaucoma, or history of ocular surgery), other systemic neurological disorders, chronic alcohol use, and conditions known to affect peripheral nerves. The study protocol was approved by the Ethics Committee of Medical University of Sofia (Approval No: 4439/05.07.2022). All participants provided written informed consent. The study complied with the ethical standards of the institutional research committee and with the Declaration of Helsinki.

### 2.2. Diagnosis of Neuropathy

**Clinical Neuropathy Assessment (Modified NDS):**

A modified Neuropathy Disability Score (NDS) was used to evaluate sensory function in the lower limbs. The assessment included examination of: Vibration perception using a 128 Hz tuning fork at the great toe; temperature sensation using a cold/hot stimulus; pinprick sensation using a disposable neurotip; ankle reflexes tested with a reflex hammer. Each parameter was graded as present/absent or present/reduced/absent for ankle reflexes and a cumulative score was calculated. NDS over 5 (modified for lower limbs) was considered positive for peripheral neuropathy. Higher scores indicated greater neuropathic impairment.

**Peripheral Neuropathy Assessment by Corneal Confocal Microscopy (CCM):**

Peripheral nerve morphology was examined using corneal confocal microscopy (Heidelberg Retinal Tomograph III with Rostock Cornea Module, Heidelberg Engineering, Germany, Heidelberg). Standardized image acquisition protocols were applied, and quantitative analysis focused on: Corneal nerve fiber density (CNFD)–fibers per mm^2^; Corneal nerve branch density (CNBD)–branches per mm^2^; Corneal nerve fiber length (CNFL)–total fiber length per mm^2^ Images were analyzed using automated software (ACCMetrics, version 2.0). Diagnostic thresholds for corneal nerve parameters were defined according to the normative values established by Malik et al. [12]

**Autonomic Neuropathy Assessment:**

Autonomic function was measured using the Cardiosys Extra system (EXPERIMETRIA, Heidelberg, Germany). Standard cardiovascular reflex tests included: Heart rate variability during deep breathing (E/I ratio); valsalva maneuver (valsalva ratio); orthostatic changes in heart rate and blood pressure. Tests were conducted under standardized resting conditions to minimize variability. A total score of ≥3 points was classified as a positive finding for autonomic neuropathy.

Combined neuropathy was defined when both DPN and autonomic neuropathy (AN) criteria were simultaneously met.

**Sudomotor Function:**

Sudomotor function was assessed using (Sudoscan^®^, Impeto Medical, Saint-Denis, France). Electrochemical skin conductance (ESC) values were obtained from hands and feet and expressed in microsiemens (µS), providing an indirect measure of sympathetic sudomotor activity and small fiber function.

### 2.3. Laboratory Investigations

**Blood Sampling:**

Venous blood samples were collected in EDTA tubes after overnight fasting. Plasma was separated by centrifugation and stored at –80 °C until analysis.

**RNA Extraction and microRNA Analysis:**

MiRNeasy/plasma kit was used for RNA extraction. MicroRNA-210-3p expression was quantified by real-time PCR (RT-qPCR) using:

- Endogenous Control (Reference Gene): An endogenous control gene with stable expression across samples was used to normalize variability. In this study, RNU6B was selected as the reference.
- Calibrator Sample: A calibrator sample was defined as the reference against which all other samples were compared.
- Ct (Cq) Value: The cycle threshold (Ct) is the PCR cycle number at which the fluorescence signal crosses a defined threshold in the exponential phase of amplification. Lower Ct values indicate higher expression levels of the target microRNA, while higher Ct values indicate lower expression.
- For qPCR we have used TaqMan microRNA assay and Universal master mix, Thermo Fisher Scientific.
- The assay ID for miR-210-3p for ordering in Thermo Fisher scientific site is 000512. The miRbase accession numbers are MI0000286 for miR-210 and MIMAT0000267 for miR-210-3p.

Real-time PCR is an exponential process in which the amplified product doubles with each cycle. During amplification, the accumulation of amplicons is monitored by the increase in reporter fluorescence. The Ct values are logarithmic and must be converted into relative linear expression values using the formulas:

ΔCt = Ct (miRNA) − Ct (reference gene)

ΔΔCt = ΔCt (control) − ΔCt (calibrator)

RQ = 2^−ΔΔCt^

where RQ stands for relative quantification (relative expression level).

RQ (Relative Quantification) represents the fold change in gene expression compared with the calibrator (which is set to 1).

Each sample was analyzed three times, and mean Ct values were used for calculations. An RQ value of >2 was considered a significant upregulation of expression, whereas an RQ value of <0.5 was interpreted as a significant downregulation.

For statistical analyses, ΔCt values were used instead of RQ values. This approach provides a more reliable statistical evaluation. ΔCt values show a more normal distribution, allow more accurate confidence interval estimation, and ensure validity in subsequent analyses. RQ values were mainly applied for interpretation and presentation of expression changes.

### 2.4. Statistical Analysis

All statistical analyses were performed using SPSS 23, IBM statistical software. Data are presented as mean ± standard deviation (SD) for normally distributed variables. Normality was assessed using the Shapiro–Wilk or Kruskal–Wallis test, and homogeneity of variances using Levene’s test. For group comparisons involving two groups, independent-samples *t*-tests were used when assumptions were met; otherwise, the Mann–Whitney U test was applied. For comparisons involving more than two groups, one-way ANOVA was performed, followed by Tukey’s HSD for post hoc pairwise comparisons. To account for multiple comparisons, post hoc *p*-values were additionally adjusted using the False Discovery Rate (FDR) procedure (Benjamini–Hochberg). Associations between continuous variables were assessed using Pearson’s correlation coefficient for normally distributed variables. A two-tailed *p*-value <0.05 was considered statistically significant. To explore independent predictors of ΔCt expression levels of microRNA-210-3p, we performed multiple linear regression analysis, adjusting for potential confounders such as age, sex, diabetes duration, HbA1c, and neuropathy measures. A two-tailed *p*-value of <0.05 was considered statistically significant for all analyses. Given the cross-sectional design, the regression models were used to assess associations rather than causality. ΔCt values represent relative miRNA expression and were treated as the dependent variable solely to explore statistical relationships with neuropathy categories. Neuropathy was not modeled as an outcome, and no causal direction was assumed. Potential confounders such as BMI, renal function (eGFR), smoking status, and use of glucose-lowering medications (GLP-1RA, SGLT-2 inhibitors, insulin, metformin) were recorded and evaluated. Due to sample size constraints, not all could be included simultaneously in multivariable models, which introduces residual confounding risk.

## 3. Results

### 3.1. Participants

Eighty patients with type 2 diabetes mellitus were included in the present study, with a mean age of 59.5 ± 7.9 years. The mean duration of diabetes was 8.8 ± 5.7 years.

### 3.2. Treatment

Only 2 patients (2.5%) were not receiving any antidiabetic medication; 33.8% were on one antidiabetic drug, 29.9% on two, 20.8% on three, 11.7% on four, and 1 patient (1.3%) was on five antidiabetic agents. As expected, the most frequently used medication was metformin (83.1%), followed by sulfonylureas (38.7%), SGLT-2 inhibitors (31.2%), GLP-1 receptor agonists (23.7%), insulin (22.4%), and DPP-4 inhibitors (10.5%).

### 3.3. Diabetes Complications

The prevalence of diabetic complications in the overall cohort was as follows: diabetic peripheral neuropathy (DPN)—57.5% (assessed via corneal confocal microscopy), autonomic neuropathy (DAN)—66.7%, nephropathy—27.3%, retinopathy—14.3%, coronary artery disease (CAD)—18.4%, history of myocardial infarction—11.8%, stroke—5.3%, and peripheral arterial disease (PAD)—5.3%.

Patients with diabetic autonomic neuropathy (DAN) were slightly older (59.4 ± 8.0 vs. 58.7 ± 9.1 years) and had a higher BMI (35.9 ± 6.4 vs. 35.2 ± 5.0 kg/m^2^) compared to those without DAN. Waist-to-hip ratio (1.01 ± 0.14 vs. 0.94 ± 0.06) was higher in patients with DAN, whereas waist-to-stature ratio was lower (0.53 ± 0.30 vs. 0.63 ± 0.16). The duration of diabetes was longer in patients with DAN (9.9 ± 6.5 vs. 8.1 ± 3.9 years). As expected, there was a trend toward older age and longer diabetes duration in patients with DAN compared to those without. No significant differences were observed between the groups regarding anthropometric parameters or other micro- and macrovascular complications.

Patients with DPN were older (60.7 ± 7.39 vs. 58.6 ± 8.17 years) and had a higher BMI (36.9 ± 6.3 vs. 34.8 ± 5.8 kg/m^2^) compared to those without DPN. Waist-to-hip ratio was similar between groups (0.98 ± 0.08 vs. 0.99 ± 0.15), while waist-to-stature ratio was slightly higher in patients with DPN (0.59 ± 0.23 vs. 0.55 ± 0.28). Diabetes duration was significantly longer in the DPN group (10.4 ± 6.0 vs. 6.8 ± 4.7 years, *p* < 0.05). HbA1c levels were also higher in patients with DPN (7.9 ± 1.4 vs. 7.3 ± 1.1%, *p* < 0.05).

### 3.4. miR-210-3p Expression

**Figure 1:**
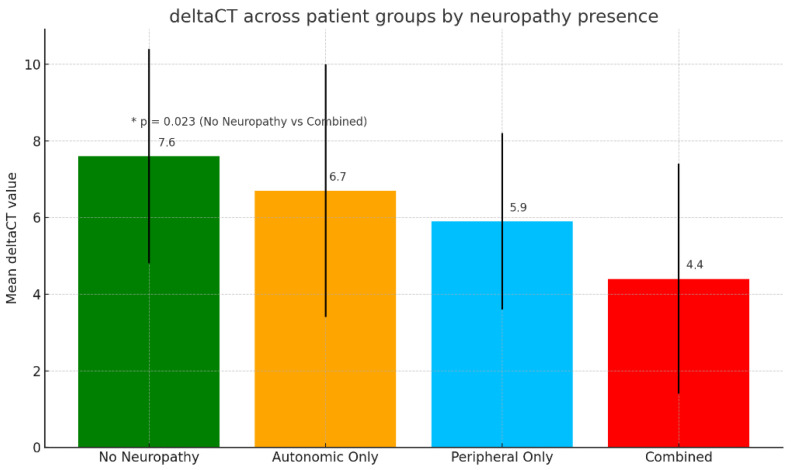
Mean ΔCt values of miR-210-3p across neuropathy categories. Bar plots show the mean ΔCt (±SD) for participants without neuropathy, with isolated autonomic neuropathy, isolated peripheral neuropathy, and combined neuropathy. ΔCt values progressively decrease from the No-Neuropathy group to the Combined-Neuropathy group, indicating higher relative miR-210-3p expression in individuals with more extensive neuropathic involvement. Only one comparison reached statistical significance (No Neuropathy vs. Combined Neuropathy, *p* = 0.023), as indicated above the bars. Statistical analysis was conducted using one-way ANOVA after verifying normality and homogeneity of variances. Tukey’s HSD was used for post hoc pairwise comparisons. To account for multiple comparisons, *p*-values were further adjusted using False Discovery Rate (FDR, Benjamini–Hochberg). An asterisk indicates a significant difference between No Neuropathy and Combined Neuropathy (* *p* = 0.023, FDR-adjusted). ΔCt values are inversely proportional to miR-210-3p expression, i.e., lower ΔCt reflects higher expression. Abbreviations: ΔCt–Delta Cycle Threshold.

Patients were divided into four groups: without neuropathy (n = 18), with only autonomic neuropathy (n = 17), with only peripheral neuropathy (n = 18), and with combined (autonomic and peripheral) neuropathy (n = 27). The highest delta Ct values (reflecting lower expression of miR-210-3p) were observed in patients without neuropathy, while the lowest delta Ct values (reflecting higher expression of mir-210-3p) were seen in those with combined neuropathy (7.6 ± 3.0 vs. 4.4 ± 2.6, *p* = 0.023). Intermediate delta Ct values were found in patients with only autonomic neuropathy (6.7 ± 3.2) and only peripheral neuropathy (5.9 ± 2.0), with no statistically significant difference between these two groups. A statistically significant difference was found between the group without neuropathy and the group with combined neuropathy (*p* = 0.023) (Figure 1).

**Figure 2:**
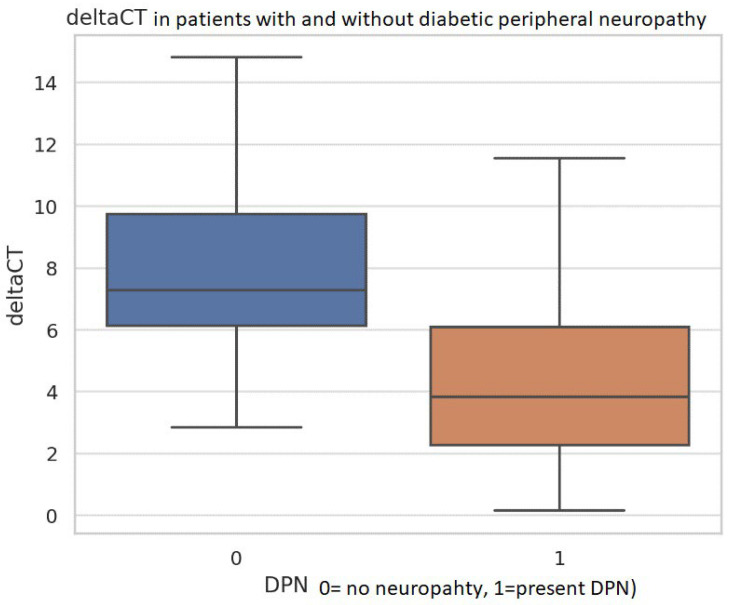
Distribution of miR-210-3p ΔCt values in patients with and without diabetic peripheral neuropathy (DPN). Box plots compare ΔCt values between individuals without DPN (0) and those with clinically confirmed DPN (1). Patients with DPN exhibit markedly lower ΔCt values, consistent with higher miR-210-3p expression. The spread of values is narrower in the DPN group, whereas the non-DPN group shows broader variability and higher median ΔCt. Outliers are shown as individual points. Normality of continuous variables was assessed. Variables that did not satisfy normality assumptions were analyzed using non-parametric methods. Exact *p*-values are reported on the figure or in the corresponding results text. To reduce the risk of false positives arising from multiple comparisons across neuropathy subgroups, *p*-values were additionally adjusted using the Benjamini–Hochberg false discovery rate (FDR) method. Abbreviations: ΔCt—Delta Cycle Threshold; DPN—Diabetic Peripheral Neuropathy.

Patients with DPN had significantly longer diabetes duration (*p* = 0.005), higher HbA1c (*p* = 0.044), elevated vibration perception threshold (*p* = 0.032), and lower delta Ct values (*p* < 0.0001) (Figure 2).

**Figure 3:**
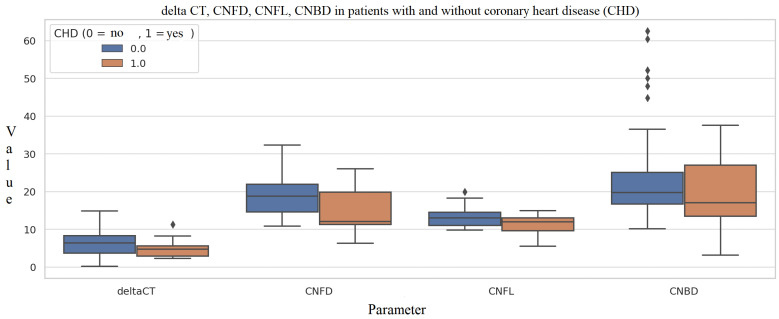
Comparison of deltaCT, CNFD, CNFL, and CNBD between patients with and without coronary heart disease (CHD). Box plots compare molecular (ΔCt) and corneal confocal microscopy (CCM) measures between individuals without CHD (0) and those with CHD (1). The CHD group shows lower ΔCt values and reduced nerve fiber parameters, although variability is substantial for CNBD due to several high-value outliers. These distributions allow visualization of potential associations between systemic vascular disease and both miRNA expression and small nerve fiber structure. Individual points represent outliers. Several variables (particularly CNBD and deltaCT) displayed non-normal distributions and heterogeneous variances tests, group comparisons were performed using the Mann–Whitney U test. Multiple parameter comparisons were corrected using the Benjamini–Hochberg false discovery rate (FDR) method to control for false positives. Abbreviations: CHD—Coronary Heart Disease; deltaCT—Delta Cycle Threshold; CNFD—Corneal Nerve Fiber Density; CNFL—Corneal Nerve Fiber Length; CNBD—Corneal Nerve Branch Density.

Patients with diagnosed coronary heart disease (CHD) had significantly lower CNFD (15.0 ± 6.1 no./mm^2^ vs. 18.8 ± 5.4, *p* = 0.029) and CNFL (19.3 ± 10.9 mm/mm^2^ vs. 23.7 ± 12.0, *p* = 0.008) compared to those without CHD (Figure 3).

**Figure 4:**
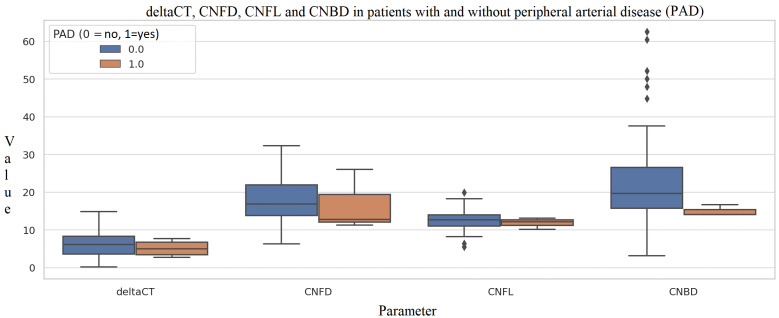
ΔCt and corneal nerve parameters (CNFD, CNFL, CNBD) in patients with and without peripheral arterial disease (PAD). Box plots depict the distribution of ΔCt and CCM-derived nerve metrics among participants without PAD (0) and with PAD (1). Compared with non-PAD individuals, those with PAD show slightly lower ΔCt values and reduced corneal nerve fiber parameters, although differences vary by metric. Outliers in CNBD reflect heterogeneity in nerve branching density.

A trend toward lower CNFD (16.7 ± 8.1 no./mm^2^ vs. 18.2 ± 5.7, *p* = 0.781) and CNFL (11.8 ± 1.5 mm/mm^2^ vs. 12.7 ± 2.6, *p* = 0.425) was also noted in patients with peripheral arterial disease, although not statistically significant (Figure 4).

Multiple regression analysis identified the presence of diabetic neuropathy as the strongest independent predictor of reduced delta Ct (β = −3.66, *p* < 0.001). Interestingly, longer diabetes duration was independently associated with an increase in delta Ct (β = +0.17, *p* = 0.019). Conversely, HbA1c did not show a statistically significant effect on delta Ct (*p* = 0.469).

## 4. Discussion

This study provides valuable insights into the differential expression of mir-210-3p in relation to the presence and type of diabetic neuropathy in patients with type 2 diabetes mellitus. The concept of “delta Ct” in gene expression analysis reflects the inverse relationship between the Ct value (cycle threshold) and the abundance of a target gene: a higher delta Ct value typically indicates lower expression of the target gene, while a lower delta Ct value signifies higher expression. Our findings reveal a statistically significant trend where the lowest delta Ct values, indicative of higher mir-210-3p expression, are observed in patients with combined neuropathy (autonomic and peripheral), compared to those without neuropathy, who exhibit the highest delta Ct values (lowest mir-210-3p expression). This suggests that mir-210-3p expression is inversely correlated with the severity of diabetic neuropathy. This study has several important limitations. Its cross-sectional design prevents causal inference between miR-210-3p expression and neuropathy, so all findings should be interpreted as associations only. The lack of a healthy, non-diabetic control group makes it unclear whether ΔCt differences reflect neuropathy, diabetes itself, or both. The modest sample size—particularly within neuropathy subgroups—limits statistical power and may affect the robustness of subgroup analyses. Finally, monocentric recruitment from a specialized endocrinology clinic may introduce selection bias and restrict the generalizability of the results to the wider T2D population.

The mean delta Ct value in the “no neuropathy” group was significantly higher than the one observed in the “combined neuropathy” group (*p* = 0.023). Intermediate delta Ct values were found in patients with only autonomic neuropathy and only peripheral neuropathy, though the difference between these two isolated neuropathy groups was not statistically significant. This gradient of mir-210-3p expression, from lowest in “no neuropathy” to highest in “combined neuropathy”, strongly suggests a potential role for this miRNA in the pathogenesis or progression of diabetic neuropathy. These findings are consistent with a study performed by Pichu et al. (2021) where an inverse relationship was reported between miR-210 and hypoxia-inducible factor 1 alpha (HIF-1α) expression in diabetic foot ulcer (DFU) patients. Since HIF-1α is crucial for angiogenesis and wound repair under hypoxic conditions, its suppression by miR-210 suggests that the microRNA may hinder proper hypoxic response and tissue recovery in chronic diabetic wounds [13]. Thus, elevated expression of miR-210-3p could contribute both to impaired wound healing in DFU and to the neurovascular dysfunction underlying diabetic neuropathy.

Further analysis, particularly through corneal confocal microscopy (CCM), strengthens this association. CCM is a non-invasive, objective imaging technique that allows for in vivo quantification of small nerve fiber morphology in the cornea, providing valuable surrogate markers for peripheral nerve health. The key parameters assessed by CCM include: Corneal Nerve Fiber Density (CNFD, Corneal Nerve Fiber Length (CNFL), Corneal Nerve Branch Density (CNBD). Over the years, many studies (Zhang et al., 2024; Ponirakis et al., 2024; Kaplan et al., 2024) have investigated the association between alterations in corneal confocal microscopy (CCM) parameters and both the development and severity of diabetic neuropathy [14,15,16]. Our results consistently demonstrate significant alterations in these CCM parameters in patients with neuropathy. Patients with DPN also had significantly longer diabetes duration, higher HbA1c, elevated vibration perception threshold, and lower delta Ct values. A direct association was found between lower delta Ct (higher mir-210-3p expression) and the presence of DPN, along with established clinical and instrumental markers of neuropathy.

Patients with diagnosed coronary heart disease (CHD) also showed significantly lower CNFD and CNFL compared to those without CHD, further implementing the simultaneous development of different diabetic complications. According to Lim et al. (2023), corneal confocal microscopy metrics were significantly lower in patients with type 1 diabetes complicated by DPN or DFU (all *p* < 0.001) compared to those without these conditions. Critically, reductions in CNFD independently predicted future cardiovascular (HR 1.67, *p* = 0.01) and cerebrovascular (HR 1.55, *p* = 0.02) events over three years, indicating that corneal nerve degeneration may serve as an early biomarker not only for peripheral neuropathy but also for broader vascular risk [17].

The observed inverse correlation between mir-210-3p expression and neuropathy severity, as evidenced by both direct assessment (delta Ct) and indirect markers from CCM, is intriguing. miR-210 is widely recognized as a “hypoxamir,” meaning its expression is often upregulated under hypoxic conditions. In the context of diabetes, chronic hyperglycemia can lead to microvascular dysfunction and localized hypoxia in various tissues, including nerves. Therefore, the elevated mir-210-3p expression in patients with more severe neuropathy (combined neuropathy and DPN), alongside the significant reductions in CNFD and CNFL, could be a response to chronic hypoxia and cellular stress in affected nerve tissues. The reduced corneal nerve parameters directly reflect the structural damage to small nerve fibers, providing a possible link to the physiological context in which mir-210-3p expression is altered.

Previous research supports the involvement of mir-210-3p in diabetic complications. Studies have shown that mir-210-3p is dysregulated in type 2 diabetes and its complications, including diabetic retinopathy. It has been implicated in regulating angiogenesis and cellular responses to hypoxia. For example, some studies suggest a protective role for miR-210 in cardiovascular homeostasis and against vascular injury. Zhou et al. (2022) demonstrate an interesting mechanism behind vascular dysfunction in T2DM by which downregulation of miR-210 in RBC (red blood cells), isolated from T2DM patients and diabetic mice induces endothelial dysfunction via interaction with vascular PTP1B (protein tyrosine phosphatase 1B) and ROS (reactive oxygen species) [18]. Another study, conducted by Song et al. suggests that microRNA-210 targeting glycerol-3-phosphate dehydrogenase controls mitochondrial bioenergetics and ROS flux and improves cardiac function in a murine model of myocardial infarction in the setting of IR (ischemia–reperfusion) injury. MicroRNA-210 deficiency significantly exaggerated cardiac dysfunction up to 6 weeks after myocardial IR in male, but not female mice and no diabetic mouse models were included [19]. A newer study, focused on mice models as well, showed that genetic overexpression of miR-210 altered the aortic transcriptome, decreasing genes in pathways involved in oxidative stress. Nitric oxide production by high glucose in endothelial cells was restored by miR-210 mimic [20].

Although these studies present compelling data regarding the possible protective effect of miR-210, the underlying biological pathways influenced by miR-210 could differ depending on the tissue or cell type being studied. Prior literature already suggests roles for miR-210 in hypoxia, diabetic complications, and vascular dysfunction, but knockdown/overexpression is mainly discussed in animal models making human relevance uncertain.

On the other hand, other research indicates that altered mir-210-3p levels can contribute to pathological processes, such as promoting obesity-induced adipose tissue inflammation and insulin resistance. Studies have shown that it is heavily involved in the polarization of adipose tissue macrophages (ATMs) and in promoting a pro-inflammatory phenotype under conditions of lipid overload and hypoxia—hallmarks of obesity. Patra et al. (2023, 2024) highlighted that miR-210-3p enhances inflammation and systemic insulin resistance by targeting the SOCS1-mediated NF-κB pathway and downregulating GLUT4, a glucose transporter critical for insulin-stimulated glucose uptake. Importantly, targeted inhibition of miR-210-3p using locked nucleic acid (LNA) technology in vivo successfully alleviated adipose tissue inflammation and improved insulin sensitivity, pointing toward a promising therapeutic strategy for managing obesity-induced insulin resistance and T2DM [10,11].

miR-210-3p is also increasingly recognized as a circulating biomarker for T2DM and its vascular complications. In a clinical study by Duisenbek et al. (2024), miR-210-3p expression was significantly higher in T2DM patients with macrovascular complications compared to non-diabetic controls, suggesting its potential role in vascular pathogenesis [21]. Similarly, Chen et al. (2022) found that elevated levels of circulating miR-210 were closely associated with obesity-related T2DM and the presence of diabetic complications, further reinforcing its role as both a biomarker and a potential mediator of disease progression [22].

The vascular implications of miR-210-3p extend into the area of diabetic microvascular complications. Yin et al. (2020) demonstrated that serum miR-210 levels were elevated in patients with diabetic retinopathy (DR) and correlated with disease severity. Functionally, miR-210 was shown to regulate vascular endothelial cell proliferation under high-glucose conditions, highlighting its potential as both a diagnostic indicator and a therapeutic target in diabetic vascular diseases [23].

The current findings, showing higher mir-210-3p expression in more severe neuropathy, in conjunction with clear evidence of structural nerve damage from CCM (reduced CNFD, CNFL, CNBD) could suggest a possible pathogenic role as elevated mir-210-3p may be involved in biological pathways relevant to nerve damage over time through complex interactions with various signaling pathways.

It is important to note that while longer diabetes duration was independently associated with an increase in delta Ct (meaning lower mir-210-3p expression), this appears to contradict the stronger association observed with the presence of neuropathy. This could suggest that while long-term diabetes might generally reduce mir-210-3p, the specific insult leading to neuropathy might trigger a localized or compensatory upregulation. The regression analyses showed that longer diabetes duration was associated with higher. This pattern may reflect a biphasic behavior in which chronic type 2 diabetes mellitus reduces baseline miRNA expression, while acute or advanced neuropathic injury triggers compensatory upregulation. This is supported by findings that miR-210 is a well-characterized “hypoxamir” that is upregulated under hypoxic conditions and that patients with diabetic foot ulcers—regions of severe hypoxia and tissue injury-exhibit elevated miR-210 levels compared to diabetic controls, strengthening the hypothesis of local upregulation in response to neuropathic or ischemic stress [13]. The strong correlation between diabetes duration and reduced CNFD, CNFL and CNBD further highlights the progressive nature of nerve damage over time, which may initially be accompanied by efforts to upregulate mir-210-3p as a compensatory mechanism, even if the overall long-term trend in the absence of acute stress is different. Further studies are needed to disentangle these complex relationships.

The lack of a statistically significant effect of HbA1c on delta Ct in the regression analysis, despite its significant correlation with DPN and CNFD, suggests that while glycemic control is crucial for managing diabetes and its complications, the direct link between acute glycemic fluctuations and mir-210-3p expression might be less pronounced than the cumulative effect of neuropathy itself or long-term diabetes duration. Evidence shows that in settings of tissue hypoxia—such as diabetic retinopathy or foot ulceration—miR-210 is elevated independent of systemic glucose levels and may even be suppressed by hyperglycemia in hypoxic tissues [23]. These findings imply that mir-210-3p may contribute to progressive nerve injury and its associated cellular stress, rather than being simply a reflection of glycemic control.

## 5. Conclusions

This study demonstrates that mir-210-3p is a possible biomarker for diabetic neuropathy, with its expression level inversely correlating with the severity of nerve damage. The consistent findings from delta Ct values, complemented by the objective structural insights from corneal confocal microscopy parameters (CNFD, CNFL, CNBD), provide a comprehensive view of small nerve fiber pathology in diabetic patients. Future research should focus on understanding the precise mechanisms by which mir-210-3p contributes to or modulates diabetic neuropathy, including its specific downstream targets and its potential as a therapeutic target for preventing or treating this debilitating complication of diabetes. The combination of gene expression analysis and non-invasive imaging techniques like CCM offers a promising method for early diagnosis, monitoring, and therapeutic intervention in diabetic neuropathy. Future research should explore the mechanistic pathways regulated by miR-210-3p and its potential as a therapeutic target in diabetic neurodegeneration.

## Data Availability

The data presented in this study are available on request from the corresponding author due to patient privacy.
